# Nematode Galectin Inhibits Basophilic Leukaemia RBL-2H3 Cells Apoptosis in IgE-Mediated Activation

**DOI:** 10.3390/ijms25137419

**Published:** 2024-07-06

**Authors:** Marta Maruszewska-Cheruiyot, Ludmiła Szewczak, Katarzyna Krawczak-Wójcik, Michael James Stear, Katarzyna Donskow-Łysoniewska

**Affiliations:** 1 Laboratory of Parasitology, General Karol Kaczkowski Military Institute of Hygiene and Epidemiology, 01-163 Warsaw, Poland; m.maruszewska@lazarski.edu.pl (M.M.-C.); ludmila.szewczak@uw.edu.pl (L.S.); katarzyna.krawczak@awf.edu.pl (K.K.-W.); 2 Department of Animal, Plant and Soil Science, Agribio, La Trobe University, Melbourne, VIC 3086, Australia; m.stear@latrobe.edu.au

**Keywords:** *Nematode* galectin, mast cells, apoptosis

## Abstract

Mast cells are essential immune cells involved in the host’s defence against gastrointestinal nematodes. To evade the immune response, parasitic nematodes produce a variety of molecules. Galectin 1, produced by *Teladorsagia circumcincta* (Tci-gal-1), reduces mast cell degranulation and selectively regulates mediator production and release in an IgE-dependent manner. To uncover the activity of Tci-gal-1, we have examined the effect of the protein on gene expression, protein production, and apoptosis in activated basophilic leukaemia RBL-2H3 cells. Rat RBL-2H3 cells were activated with anti-DNP IgE and DNP-HSA, and then treated with Tci-gal-1. Microarray analysis was used to examine gene expression. The levels of several apoptosis-related molecules and cytokines were determined using antibody arrays and ELISA. Early and late apoptosis was evaluated cytometrically. Degranulation of cells was determined by a β-hexosaminidase release assay. Treatment of activated RBL-2H3 cells with Tci-gal-1 resulted in inhibited apoptosis and decreased degranulation, although we did not detect significant changes in gene expression. The production of pro-apoptotic molecules, receptor for advanced glycation end products (RAGE) and Fas ligand (FasL), and the cytokines IL-9, IL-10, IL-13, TNF-α, and IL-2 was strongly inhibited. Tci-gal-1 modulates apoptosis, degranulation, and production of cytokines by activated RBL-2H3 cells without detectable influence on gene transcription. This parasite protein is crucial for modulation of the protective immune response and the inhibition of chronic inflammation driven by mast cell activity.

## 1. Introduction

Mast cell activation by IgE is one of the most important immune mechanisms against gastrointestinal nematode parasites, including *Teladorsagia circumcincta* [1]. Mast cells play an essential role in the fight against nematodes. The FcεRI receptor binds IgE, which can then recognise nematode antigens, resulting in mast cell degranulation. Histamine, serotonin, heparin, and neutral proteases, as well as cytokines, chemokines, lipid-derived mediators, and growth factors, are all found in mast cell cytoplasmic granules, and are released through degranulation [2]. In response to parasite invasion, mast cells generate cytokines that stimulate the activity of innate immune cells, eosinophils and neutrophils, as well as cells engaged in adaptive immune responses, such as dendritic cells and T cells. There are further physiological mechanisms that are initiated by mast cell-produced mediators that prevent or reduce parasite invasion. Mast cell-derived mediators also stimulate smooth muscle, promote helminth expulsion, and induce mucus production by epithelial cells [3]. However, the mast cell response is slow to develop, and expulsion after a primary infection of *T. circumcincta* is uncommon, even in hosts with high levels of IgE. This results in a significant problem, as the prevalence of this parasite among farm animals in many regions of the world causes significant economic losses [4]. The parasite is becoming more resistant to anthelmintics, making nematode control much more challenging [5]. Understanding the immune response to nematodes and how *T. circumcincta* responds to host defence is critical for identifying and developing new drugs and treatments against parasites.

Parasitic nematodes produce various molecules to modulate host immune responses. Galectins are one group of these modulators. They are proteins that bind β-galactosides, and they have been observed in a variety of animal groups, including mammals [6]. Galectins influence a variety of biological activities, including the cell cycle and immune cell proliferation [7]. Changes in the level of host galectins during infection suggest a significant role of galectins in the course of parasite infections. *T. circumcincta* infection leads to the production of sheep galectins −14 and −15 [8]. Parasitic nematodes produce their own galectins to alter the host galectin effect during infection [6]. Galectins have been identified in various nematode species, such as *Haemonchus contortus, T. circumcincta*, *Strongyloides ratti, Ancylostoma caninum, A. cantonensis, A. costaricensis, Diactyocaulus viviparus, Heligmosomoides polygyrus, Nipostrongylus brasiliensis*, *Onchocerca volvulus, Toxocara canis, Trichostrongylus colubriformis,* and *Trichinella spiralis* [9]. Furthermore, parasite-derived galectins regulate the survival of immune cells. Galectin-1 from *Angiostrongylus cantonensis* induces macrophage apoptosis [10]. Galectins, together with molecules from the venom allergen family, are major components of *T. circumcincta* larvae excretory–secretory products [11].

Galectin 1 produced by *T. circumcincta*, (Tci-gal-1) has strong immunomodulatory capabilities and exhibits sequence similarities in the carbohydrate binding site with human Gal-3 and Gal-9 [6]. Previously conducted experiments on rat basophilic leukaemia cells (RBL-2H3 cell line), which is a well-documented model of IgE-dependent activation of mucosal mast cells [12], indicated that Tci-gal-1 affects RBL-2H3 cell activity by influencing FcεRI-dependent mast cell activation. *T. circumcincta* galectin reduced IgE antibody binding to mast cell surface, inhibited degranulation, and selectively influenced mediator release [13]. The objective of this research was to investigate how Tci-gal-1 affects gene expression and protein production in activated RBL-2H3 cells. The results show that Tci-gal-1 inhibits apoptosis in activated RBL-2H3 cells. We then investigated expression of selected genes involved in apoptosis, but we did not detect a strong influence on gene expression.

## 2. Results

### 2.1. Tci-Gal-1 Influences Expression of Genes Encoding Proteins Related to Apoptosis

In order to determine changes in gene expression in RBL-2H3 cells under Tci-gal-1 influence, we evaluated the gene expression level of RBL-2H3 cells activated with anti-DNP IgE and DNP-HSA (Ag), and then treated with Tci-gal-1, compared to cells treated with IgE and HSA alone using the microarray method. From this, 196 genes showed a greater than 1.5-fold altered expression level. Based on increased fold change (min. 2), we obtained a list of 94 genes with different expression levels. However, the False Discovery Rate (FDR) analysis showed that there were no statistically significant changes in gene expression amongst the genes studied. Hence, we decided to focus on genes that may be important in future studies, taking into account the fact that there are generally no transcriptional changes in our experimental setting.

**Figure 1:**
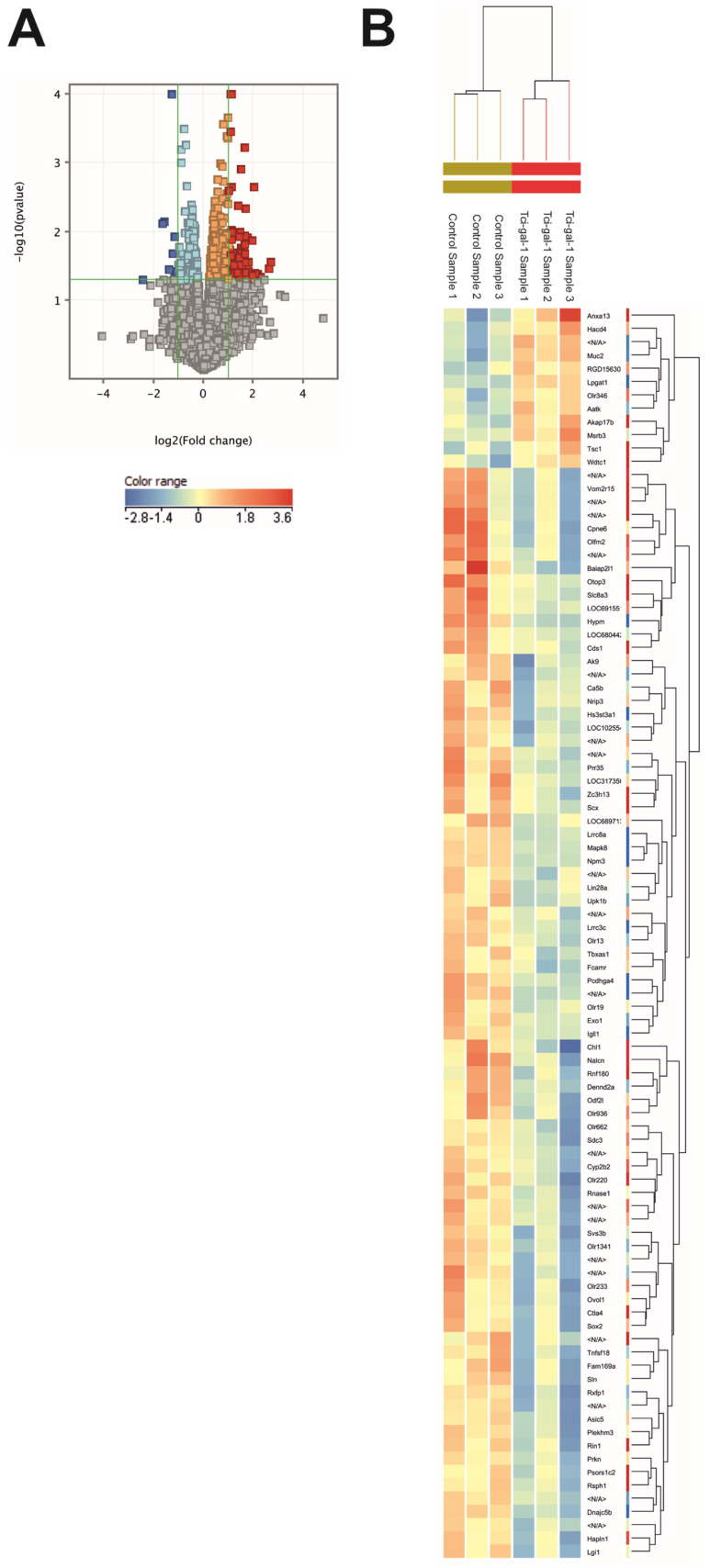
Tci-gal-1-induced gene expression changes in activated RBL-2H3 cells. Gene expression was evaluated after 60 min of treatment of activated RBL-2H3 cells with Tci-gal-1 by microarray. Upregulated and downregulated genes were selected using Genespring GX software version 14.9. (**A**) The volcano plot shows *p*-value versus fold change in genes regulated by Tci-gal-1. (**B**) The genes and experimental variants are clustered based on the similarities of their normalized expression. The volcano plot and heatmap were created using Genespring GX software. Control—activated RBL-2H3 cells; Tci-gal-1—activated RBL-2H3 cells treated with Tci-gal-1.

A volcano plot presenting the relationship between the *p*-value and fold change in all evaluated genes is shown in Figure 1A. The changes are also presented as a heatmap (Figure 1B). A list of the genes with a fold change greater than 2 has been included in the supplement (Supplementary S1A,B), in which there were 82 upregulated and 12 downregulated genes. Among genes with a fold change greater than 2, 12 were unidentified. The two most highly upregulated genes were genes encoding Copine 6 and BAI1-associated protein 2, which is like 1 with a fold change over 6. Another five genes were upregulated over 4-fold, as follows: an unknown gene (A_64_P131482), *olfactomedin 2*, *cell adhesion molecule L1-like*, another unknown gene (A_64_P106893), and *huntingin-interacting protein M* gene. The most downregulated gene was *annexin A13*. Based on the list of genes, we selected genes that encode proteins of the greatest importance for cell survival and apoptotic processes. We focussed on apoptosis because it plays a key role in mast cell-mediated immunomodulation. The selected genes are listed in Table 1. In addition to those previously mentioned, these include *Ctla4* (fold change 2.9), *Cyp2b2* (fold change 2.3), *Mapk8* (fold change 2.1), *Tnfst18* (fold change 3.1), and *Tsc1* (fold change 2.2).

### 2.2. Tci-Gal-1 Inhibits Apoptosis of Activated RBL-2H3 Cells

**Figure 2:**
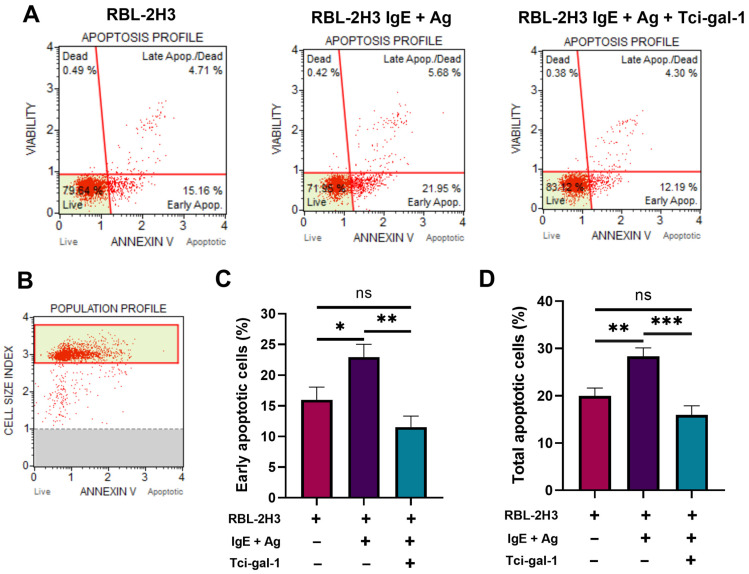
Tci-gal-1 affects apoptosis of activated RBL-2H3 cells. Apoptosis was evaluated after 60 min culture of activated RBL-2H3 cells in the presence of Tci-gal-1 using The Muse Annexin V & Dead Cell Assay (Muse Cell Analyzer; Merck Millipore, Burlington, MA, USA). (**A**) Representative plots of apoptosis profile of RBL-2H3 cells. (**B**) Population profile of RBL-2H3 cells in apoptosis analysis. (**C**) Percentage of early apoptotic RBL-2H3 cells. (**D**) Percentage of total apoptotic RBL-2H3 cells. Data are expressed as mean percentage of cells ± SD data from three independent experiments, ns- not significant, * *p* < 0.05, ** *p* < 0.001, and *** *p* < 0.0001, compared to control.

In order to determine the effect of Tci-gal-1 on apoptosis of RBL-2H3 cells, the percentage of early apoptotic, late apoptotic, and necrotic cells was cytometrically assessed in activated RBL-2H3 cells treated with Tci-gal-1. Activation of RBL-2H3 cells with IgE and antigen resulted in an increased percentage of early apoptotic cells, and the total number of early apoptotic, late apoptotic, and necrotic cells. The addition of Tci-gal-1 reduced the number of both early and late apoptotic cells (Figure 2).

### 2.3. Tci-Gal-1 Inhibits Degranulation of Activated RBL-2H3 Cells

**Figure 3:**
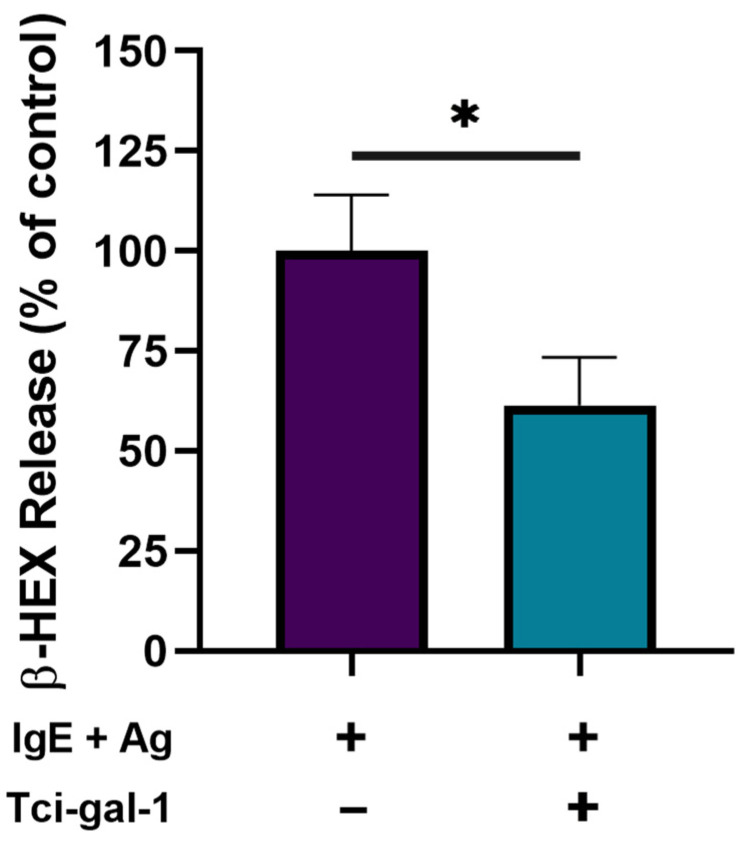
Tci-gal-1 affects degranulation of activated RBL-2H3 cells. Degranulation level was evaluated in medium collected after 60 min of activated RBL-2H3 cells culture in Tci-gal-1 presence using the β-hexosaminidase (B-HEX) assay. The amount of the enzyme released into the supernatants is expressed as the percentage of total β-hexosaminidase in the cells ± SD calculated from three independent experiments. * *p* < 0.05.

The influence of Tci-gal-1 on activated RBL-2H3 cells’ degranulation was evaluated based on lysosomal enzyme β-hexosaminidase release. The addition of Tci-gal-1 to activated RBL-2H3 cells significantly reduced degranulation (Figure 3).

### 2.4. Tci-Gal-1 Inhibits Expression of Molecules Involved in Apoptosis Induction by RBL-2H3 Cells

**Figure 4:**
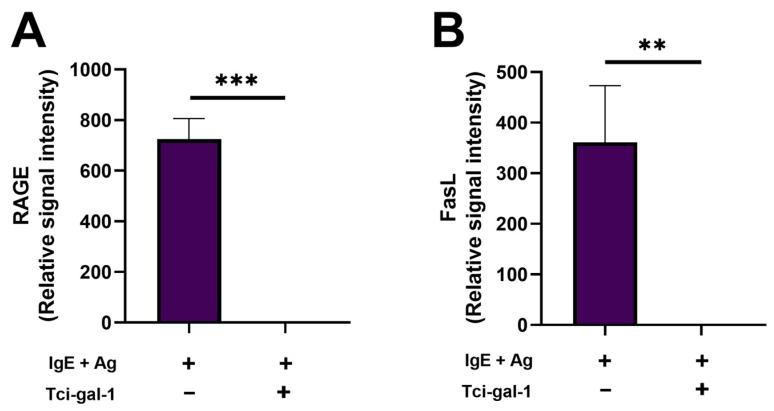
Production of molecules involved in apoptosis induction by RBL-2H3 cells under Tci-gal-1 influence. Level of molecules was evaluated in medium collected after 60 min of activated RBL-2H3 cells culture in Tci-gal-1 presence using Antibody Array. Mean relative intensity of RAGE (**A**) and FasL (**B**). Data are presented as mean relative intensity ± SD of two technical replicates. Data are representative of three independent experiments. ** *p* < 0.01 and *** *p* < 0.001.

Various molecules are involved in the induction of apoptosis. To evaluate how Tci-gal-1 inhibits apoptosis of RBL-2H3, the level of receptor for advanced glycation end products (RAGE), which is strongly involved in the induction of apoptosis, was measured. Production of RAGE was not detected in the culture medium of activated RBL-2H3 cells treated with Tci-gal-1. In contrast, untreated activated RBL-2H3 cells showed production of the molecule. To examine the potential effect of Tci-gal-1 on the influence of mast cells on the survival of other cells, the level of FasL (the ligand for the Fas receptor that has a central role in regulation of programmed cell death) was examined. Similarly to RAGE, a culture of RBL-2H3 cells in the presence of Tci-gal-1 inhibited the production of FasL (Figure 4).

### 2.5. Tci-Gal-1 Modulates Production of Cytokines Related to Mast Cell Activation

**Figure 5:**
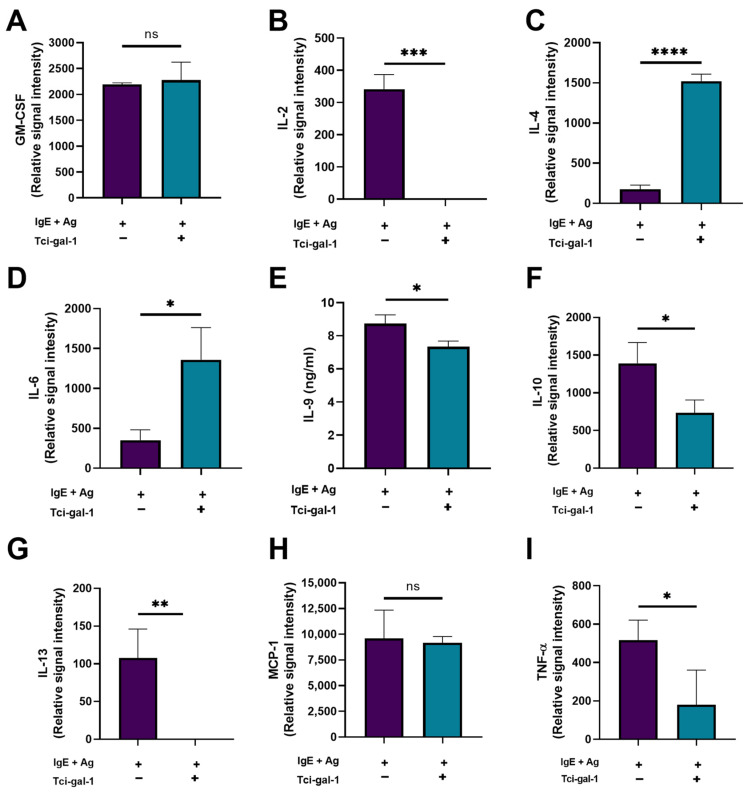
Production of cytokines by RBL-2H3 cells under the influence of Tci-gal-1. The level of cytokines was evaluated in medium collected after 60 min in culture of activated RBL-2H3 cells in the presence of Tci-gal-1, using Antibody Array or ELISA. Mean relative intensity or concentration of GM-CSF (**A**), IL-2 (**B**), IL-4 (**C**), IL-6 (**D**), IL-9 (**E**), IL-10 (**F**), IL-13 (**G**), MCP-1 (**H**), TNF-α (**I**). Data are presented as mean relative intensity ± SD of two technical replicates. Data are representative of three independent experiments, ns—not significant,* *p* < 0.05, ** *p* < 0.01, *** *p* < 0.001, and **** *p* < 0.0001.

Cytokines regulate a variety of biological processes, including cell activity, proliferation, differentiation, and survival. To assess how Tci-gal-1 impacts cytokine production, we examined the quantities of cytokines released by RBL-2H3 cells, including GM-CSF, IL-2, IL-4, IL-6, IL-9, IL-10, IL-13, MCP-1, and TNF-α. Tci-gal-1 inhibited the production of IL-2, IL-9, IL-10, IL-13, and TNF-α in RBL-2H3 cells. Tci-gal-1-induced increased production of IL-4 and IL-6 by RBL-2H3 cells (Figure 5). Differences in levels of GM-CSF and MCP-1 were not statistically significant.

## 3. Discussion

Apoptosis is controlled, programmed cell death which helps to delete unnecessary cells in a safe way. Apoptosis is important in regulating the immune response, and this makes apoptosis one of the mechanisms modulated by parasites. The aim of this modulation is to influence the course of the antiparasitic immune response, and to increase the chance of parasite survival and reproductive success [14]. Nematodes can influence apoptosis of immune cells. *H. polygyrus* antigen, which contains, among others molecules, galectin, decreases the apoptosis of CD4 T cells [15]. Proteins excreted/secreted by *Onchocerca volvulus*, a parasitic nematode that causes river blindness in humans, induce apoptosis of spleen cells [16], and infection with *Trichuris muris* is associated with increased apoptosis of intestinal epithelial cells [17].

Here, we found that *T. circumcincta* galectin Tci-gal-1 inhibits apoptosis of RBL-2H3 cells. The percentage of cells with initiated apoptotic processes, as well as during apoptosis, was decreased. This result was in line with the decreased degranulation and level of expression of a receptor involved in apoptosis induction (RAGE). Further, Tci-gal-1 inhibits FasL production by mast cells. The modulation of apoptosis by human galectins has been demonstrated. Galectin-1, -2, -3, -8, and -9 induce T cell death in an apoptosis-dependent manner [18,19,20,21]. However, intracellular Gal-3 suppresses T cell apoptosis [22]. Human Gal-3, which shows structural similarity to Tci-gal-1, induces death of mast cells with RAGE participation [23], whereas Tci-gal-1 inhibits the expression of RAGE, which translates into inhibition of the apoptosis process in mast cells. This indicates that the parasite galectin has different effects to that of the host Galectin-3, and it may compete with host galectin.

Apoptosis of immune cells can be mediated by the intrinsic apoptotic pathway or the extrinsic pathway, which are controlled by signals delivered from death receptors, such as Fas [24]. Tci-gal-1 inhibits the production of FasL, the most significant molecule involved in the induction of apoptosis. Tci-gal-1, through mast cell-inhibited production of FasL, may modulate the viability of other immune cells in the microenvironment. This mechanism could be crucial in inhibiting chronic inflammation, which is driven by mast cell activity.

These results were accompanied by inhibited degranulation of activated RBL-2H3 cells. It is consistent with our previous findings that Tci-gal-1 suppresses the release of mediators generated by mast RBL-2H3 cells stimulated in the presence of sheep serum from nematode infected sheep [13].

Activated mast cells produce a range of cytokines which influence other immune cells and play an important part in the establishment of antiparasitic immune responses. Most of them regulate the growth, proliferation, and activity of immune cells, including mast cells. Some cytokines can also play a role in the induction or inhibition of apoptosis. Here, increased RBL-2H3 cell survival during Tci-gal-1 exposure was associated with inhibited cytokine production, including IL-9, IL-10, IL-13, TNF-α, and IL-2. IL-9 contributes to the development of an antiparasitic immune response by inducing the synthesis of antibodies, especially IgE, and increasing the number of mast cells. Mast cells express the receptor for IL-9. In addition, IL-9 contributes to intestinal nematode expulsion [25]. IL-10 stimulates FcɛRI expression and regulates cytokine production by mast cells [26]. IL-13 induces mucous production, class switching of B-cell antibodies from IgM to IgE, and an increase in blood eosinophils [27]. The TNF cytokine family plays a critical role in the development of inflammatory responses. TNF-α binds to TNF receptors (TNFR) and activates signalling pathways in cells, leading to apoptosis [28]. Therefore, Tci-gal-1 may promote mast cell survival and suppress inflammation through TNF-α level reduction. In addition, it could inhibit the antiparasitic response.

Cytokines whose levels were increased were IL-4 and IL-6. IL-4 is classified as a Th2-type cytokine and mediates the development of Th2-cell differentiation. Th2-related immune responses affect the survival of the parasite and induce tissue repair processes [29]. IL-4 promotes mast cell survival and proliferation. The cytokine enhances the level of FcεRI on differentiated human mast cells [30]. Mast cells from Gal-3-deficient mice release less IL-4 during mast cell degranulation [31]. IL-6 generally shows proinflammatory activity; however, it is critical for mast cell survival [32], which suggests that Tci-gal-1 induces longer activity in mast cells and inhibits apoptosis of RBL-2H3 cells. At the same time, the levels of GM-CSF and MCP-1 did not change under the influence of Tci-gal-1.

Microarray analysis of gene expression based on FDR did not show statistically significant differences between activated RBL-2H3 cells and activated RBL-2H3 cells treated with Tci-gal-1. However, reduced expression of genes involved in the apoptotic pathway (*Annexin A13, Tsc1, Aatk*) was observed. Annexins are a protein family that can bind to specific membrane phospholipids in a Ca^2^+-dependent way, linking Ca^2^+ signalling to membrane activities [33]. Annexin A13, comparable to other annexin family members, binds to apoptotic cells. According to Rosenbaum et al. [34], this mechanism, like opsonin, assists in the detection of dying cells. The *Aakt* gene was downregulated by Tci-gal-1. Apoptosis-associated tyrosine kinase (Aakt) is a protein with well-documented involvement in inhibiting cell division and migration and inducing the process of apoptosis [35]. A decreased level of expression of genes encoding important elements involved in apoptosis can indicate the signal pathways involved in Tci-gal-1-dependent apoptosis inhibition in mast cells. The small number of genes with a changed expression level in activated RBL-2H3 cells treated with Tci-gal-1 could be because cells used previously produced preformed molecules for the immediate response. Another explanation could be the study design. Different time of stimulation with Tci-gal-1 could be necessary to induce changes in transcription. Another possible explanation is that the large number of examined genes reduced the statistical power.

These results clearly demonstrate that Tci-gal-1 modulates RBL-2H3 cells’ activity. Although RBL-2H3 cells apoptosis is inhibited, Tci-gal-1 influences the synthesis of critical cytokines involved in the development of the antiparasitic immune responses. In our previous studies, we observed that nematode galectin decreased the release of LCT-4 and β-hexosaminidase, but not MMP-9 [13]. The effect of Tci-gal-1 on RBL-2H3 cells’ activity is selective; it does not completely inhibit the production of all molecules influencing the immune response, but modulates key molecules in parasite survival while not interfering with other processes critical to the host’s health.

## 4. Materials and Methods

### 4.1. Expression and Purification of Recombinant Tci-Ga1-1

The expression and purification of recombinant Tci-gal-1 were described previously [13]. Briefly, Tci-gal-1 *P. pastoris* cells were inoculated into 10 mL of yeast extract peptone dextrose (YEPD) seed culture (1% (*w*/*v*) yeast extract, 2% (*w*/*v*) peptone, 2% (*w*/*v*) dextrose, and 100 μg/mL ZeocinTM), and cultured at 28 °C for 48 h in a shaking incubator (180 rpm) (NB-205LF, N-BIOTEK, Seoul, Republic of Korea). Expression cultures were set up with 10 mL of seed culture added to 400 mL of buffered methanol complex (BMMY) medium (1% (*w*/*v*) yeast extract, 2% (*w*/*v*) peptone, 1 % (*w*/*v*) yeast nitrogen base, 100 mM potassium phosphate pH 6.0, 100 μg/mL ZeocinTM, and 0.5% (*v*/*v*) methanol). Cells were cultured for 96 h at 28 °C while shaking (160 rpm), with 0.5% (*v*/*v*) methanol added every 24 h. Cells were centrifuged at 6000× *g* for 15 min, and the supernatant was dialysed in dialysis tubing (Sigma-Aldrich, New York, NY, USA) into starter buffer (5 mM NaH_2_PO_4_ pH 7.6, 50 mM NaCl and 2 mM imidazole) at 4 °C for 48 h, with at least three buffer changes. Dialysed media were concentrated overnight with polyethylene glycol (PEG) 8000 (Astral Scientific, Taren Point, Australia). Nickel-nitrilotriacetic acid (Ni-NTA) affinity chromatography was used to purify His-tagged Tci-gal-1 from dialysed supernatant. Ni-NTA agarose resin (2 mL) (His60 Ni Superflow Resin, Takara Bio Inc., Clayton, Australia) was added to a purification column and equilibrated with 10 bed volumes of starter buffer. Culture supernatants were added to the column. The resin was then washed (starter buffer containing 20 mM imidazole). Elution of bound proteins was performed using elution buffer (starter buffer containing 250 mM imidazole), followed by elution buffer containing 500 mM imidazole. The elution fractions were pooled and buffer-exchanged into storage buffer (25 mM NaH_2_PO_4_ and 250 mM NaCl, pH 7.6). Tc-gal-1 synthesis was determined by 12% (*w*/*v*) SDS-PAGE, followed by circular dichroism (CD) spectroscopy and mass spectrometry [13].

### 4.2. RBL-2H3 Cell Culture and Tci-Gal-1 Stimulation

The rat basophilic leukaemia RBL-2H3 cell line was obtained from the American Type Culture Collection, CRL-2256. RBL-2H3 cells were cultured in Dulbecco’s modified Eagle’s medium (Biowest, Nu-aillé, France), supplemented with 15% foetal bovine serum (FBS) (Biowest, Nu-aillé, France), penicillin/streptomycin (100 U/mL Biowest, Nu-aillé, France), and L-glutamine (2 mM, Biowest, Nu-aillé, France) in a humidified atmosphere at 37 °C and 5% CO_2_. Then, 20,000 RBL-2H3 cells per well were seeded into 96-well plates a day before the assay and cultured at 37 °C in a humidified CO_2_ incubator. Each cell layer was washed once with assay buffer (Hanks’ balanced salt solution containing 20 mM HEPES and 1 mg/mL BSA), and then activated for 60 min with 0.1 mg/mL anti-DNP IgE (Mouse IgE anti-2,4-dinitrophenol (DNP) antibody, clone Spe7; Sigma-Aldrich, St. Louis, MO, USA;Cat# D8406, RRID:AB_259249), and 0.02 mg/mL DNP-HSA (DNP-conjugated human serum albumin, Sigma-Aldrich, St. Louis, MO, USA). Tci-gal-1 was added after anti-DNP IgE for 60 min. Control wells that had no Tci-gal-1 were added. Collected culture media and RBL-2H3 cells were immediately analysed or stored at −80 °C.

### 4.3. Apoptosis Assay

To test apoptosis, RBL-2H3 cells were washed twice in ice-cold PBS pH 7.2. Apoptosis was examined using The Muse Annexin V & Dead Cell Assay (Merck Millipore, Burlington, MA, USA), which allowed a quantitative analysis to be made of live, early, and late apoptosis, and cell death antibodies based on Annexin V and 7-Aminoactinomycin (7-AAD) presence in a Muse Cell Analyzer (Merck Millipore, Burlington, MA, USA). All procedures were performed according to the manufacturer’s guidelines.

### 4.4. Degranulation Assay

To evaluate degranulation of activated RBL-2H3 cells under influence of Tci-gal-1, a β-hexosaminidase release assay was conducted. First, 50,000 RBL-2H3 cells in 200 μL were seeded into each well of a 96-well plate, a day before the assay, and cultured as above at 37 °C in a humidified incubator (5% CO_2_, 95% air). Each well was washed once with DMEM medium, and then RBL-2H3 cells were activated as above. Control wells had no Tci-gal-1 added. For the β-hexosaminidase (β-HEX) assay, supernatants were collected 60 min after the addition of Tci-gal-1. The remaining cells were lysed with 0.1% Triton X-100 (Chempur, Piekary Śląskie, Poland), and the lysate was collected. The amount of β-HEX was determined by measuring the enzyme activity. Briefly, 30 μL of each supernatant or cell lysate was mixed with 50 μL of 1.3 mg/mL 4-nitrophenyl 2-acetamido-2-deoxy-β-D-glucopyranoside (Sigma-Aldrich, St. Louis, MO, USA), dissolved in 100 mM citrate buffer (pH 4.5), and then incubated at 37 °C for 60 min. The reaction was terminated by the addition of 200 µL of 0.1 M Na_2_CO_3_/NaHCO_3_ (pH 10.0), and the optical density was read at 405 nm using a Synergy™ H1 Microplate Reader (BioTek, Winooski, VT, USA). The amount of the enzyme released into the supernatants was expressed as the percentage of total β-hexosaminidase in the cells.

### 4.5. Antibody Array

Cytokine levels (Activin A, Agrin, Thymus Chemokine-1, B7-2/CD86, CINC-1, CINC-2α, CINC-3, CNTF, Fas Ligand, Fractalkine, GM-CSF, ICAM-1, IFN-γ, IL-1 R6, IL-10, IL-13, IL-1α, IL-1β, IL-2, IL-4, IL-6, Leptin, LIX, L-Selectin, MCP-1, MIP-3α, MMP-8, β-NGF, PDGF-AA, Prolactin R, RAGE, TIMP-1, TNF-α, VEGF) produced by RBL-2H3 cells in culture medium were analysed using the Cytokine Array (Rat Cytokine Antibody Array; Abcam, Cambridge, UK, ab133992), according to the manufacturer’s instructions. The exposure time was 5 min, and the examination was carried out in 20 min since chemiluminescence signals degrade over time. The Syngene G-Box was used to scan the membranes, and the signal values were evaluated using Image J software version 1.53h. The array’s internal positive and negative controls were used to normalize the signals.

### 4.6. ELISA

The culture supernatants were collected 60 min after stimulation to measure the level of IL-9. A Rat IL-9 ELISA Kit (E0138Ra) (limits of detection 0.53 ng/mL; Bioassay Technology Laboratory, Shanghai, China) was performed on triplicate samples according to the manufacturer’s instructions, except that 20 mM lactose was added to avoid interference with the ELISA by nematode galectin carried over from the supernatant. The absorbances were measured using the Synergy™ H1 Microplate Reader (BioTek, Winooski, VT, USA).

### 4.7. Gene Expression Microarray

RNA was extracted from cells using Maxwell^®^ RSC simply RNA Blood Kit on a Maxwell RSC Instrument (Promega, Madison, WA, USA) according to the manufacturer’s instructions. Concentration and quality of RNA were assessed electrophoretically and by a Nabi UV/Vis Nano spectrophotometer (MicroDigital Co., Ltd.; Seoul, Republic of Korea). Rat GE 4x44K v3 Microarray Kit (Agilent Technologies, Santa Clara, CA, USA) was used to analyse a panel of mast cell genes. A spike mix prepared with RNA Spike-In Kit, One-Color (Agilent Technologies, Santa Clara, CA, USA) was added to each RNA sample to provide quality controls. Then, 100 ng of RNA was labelled and amplified using the Low Input Quick Amp Labeling Kit, One-Color (Agilent Technologies, Santa Clara, CA, USA) to obtain cRNA. The cRNA samples were purified using the RNeasy Mini Kit (Qiagen, Hilden, Germany). Then, hybridization samples were prepared using the Gene Expression Hybridization Kit (Agilent Technologies, Santa Clara, CA, USA). Hybridization was conducted on the microarray slide, situated in a Hybridization Oven Rotator Rack (Agilent Technologies, Santa Clara, CA, USA). Slides were scanned using a microarray scanner, NimbleGen MS200 (Roche, Switzerland). Data were extracted using Feature Extraction software version 9.5.3.1. (Agilent Technologies, Santa Clara, CA, USA). To evaluate the reproducibility and reliability of microarray experiments, a quality control report was generated for each sample.

### 4.8. Statistical Analysis

All experiments and tests were performed in triplicate to ensure reliable results. The significance of differences was defined with Student’s *t*-test (two-tailed unpaired), or the Mann–Whitney test (GraphPad Software Inc. version 8.4.3; Boston, MA, USA). The data were presented as mean ± SD. A *p*-value of <0.05 was considered to be statistically significant. The microarray data were analysed using Genespring GX software version 14.9, and the fold change (Fc) was greater than 2. A Moderated T-Test was used to calculate *p*-values for genes that were up- and downregulated. Filtered gene lists were generated for expression changes greater than 2-fold and a *p*-value less than 0.05. To avoid false positive associations due to multiple comparisons, the False Discovery Rate (FDR) was calculated using the MULTITEST procedure in SAS version 9.4 (SAS Institute, Cary, NC, USA)

## 5. Conclusions

Nematode galectin Tci-gal-1 inhibits apoptosis of RBL-2H3 cells. The parasite possibly maintains the viability of cells that inhibit the antiparasitic response. Classically, activation of mast cells induces mast cell apoptosis, indicating that mast cells can be eliminated soon after activation. The inhibition of mast cell apoptosis could be a crucial mechanism to modulate the immune response of the host.

Further, Tci-gal-1 inhibits mast cell release of FasL, a molecule that plays a central role in apoptosis. This mechanism could be crucial in inhibiting chronic inflammation, which is driven by mast cell activity. An improved understanding of the molecules produced by intestinal nematodes such as Tci-gal-1 could lead to better methods of parasitic disease control, and may lead to immunomodulators that can treat autoimmune, inflammatory, or allergic diseases.

## Figures and Tables

**Table 1:** List of genes regulated in RBL-2H3 by Tci-gal-1 that are important for mast cell apoptosis.

Gene	Description	Regulation	Fold Change	Adjusted *p*-Value
*Aatk*	apoptosis-associated tyrosine kinase	down	2.2	0.0117
*Anxa13*	annexin A13	down	5.4	0.0498
*Baiap2l1*	BAI1-associated protein 2-like 1	up	6.2	0.0348
*Tsc1*	TSC complex subunit 1 (Tsc1)	down	2.2	0.049

## Data Availability

Data supporting the conclusions of this article are included within the article.

## Supplementary Materials

The following supporting information can be downloaded at: https://www.mdpi.com/article/10.3390/ijms25137419/s1.
